# Who responds? An examination of response rates to a national postal survey of Aboriginal and Torres Strait Islander adults, 2018-2019

**DOI:** 10.1186/s12874-020-00970-8

**Published:** 2020-06-10

**Authors:** Alyson Wright, Katherine Ann Thurber, Mandy Yap, Wei Du, Emily Banks, Jennie Walker, Faye Irwin, Will Sanders, Raymond Lovett

**Affiliations:** 1 National Centre for Epidemiology and Population Health, Research School of Population Health, Australian National University, Canberra, Australia; 2 Centre for Aboriginal Economic Policy Research, College of Arts and Social Sciences, Australian National University, Canberra, Australia

**Keywords:** Indigenous, cohort, recruitment, postal surveys, response rates, Aboriginal, Torres Strait Islander

## Abstract

**Background:**

Evidence on the effectiveness of postal recruitment methods for Indigenous peoples is lacking. Mayi Kuwayu, the National Study of Aboriginal and Torres Strait Islander Wellbeing, uses multi-staged sampling. We aimed to test postal surveys as a primary recruitment method, analysing preliminary response rate data to inform the Study’s ongoing sampling approach.

**Methods:**

Twenty thousand adults aged ≥16 years were sampled from Aboriginal and Torres Strait Islander people enrolled in the Medicare Australia Enrolment Database. We calculated response rates at 4 and 15 weeks, overall and by age group, gender, state/territory and remoteness.

**Results:**

The overall response rate was 2.3% (*n* = 456/20000). Highest response rates were observed among males and females ≥50 years from major cities (6.0, 95%CI 4.4–7.9 and 5.5%, 4.1–7.2, respectively) and regional areas (6.0%, 4.6–7.6 and 6.2%, 4.9–7.7, respectively). Younger age groups and remote areas had lower response rates; all remote age groups < 50 years had a response rate ≤ 0.6%. While most participants responded on the paper surveys, online responses were more common among younger age groups and, respondents with higher education levels and whose first language was not English.

**Conclusion:**

Using a postal survey, we observed response rates of ≥5.5% among older Aboriginal and Torres Strait Islander adults in major cities and regional areas; response rates were lower in other groups. A two-stage postal distribution approach provided an opportunity to adapt sampling approaches to different demographic groups. Based on initial response rates, the sampling strategy was revised to send postal surveys to groups with higher response rates groups and focus field recruitment strategies on low response groups.

## Background

It is internationally recognised that improvements in data systems are needed to support action and progress in Indigenous peoples’ health outcomes [1, 2]. This includes the need for longitudinal data on wellbeing measures that are defined by Indigenous people. These calls also recognise the importance of Indigenous peoples’ role in determining data processes, including the sampling approaches applied. Longitudinal (cohort) studies can and do contribute to the growing evidence base on Indigenous perspectives on health and wellbeing but to date Indigenous cohort studies in health have been small in scale or confined to geographic areas or language groups.

Postal surveys have been routinely used as a primary method of recruitment for large-scale national cohort studies and are a particularly efficient method for dispersed populations [3]. Yet, postal response rates are unknown for most Indigenous populations, because cohort studies in these populations have tended to opt for other recruitment approaches [4–6]. Examination of response rates is needed to determine if postal surveys are an effective mechanism for the collection of large scale, longitudinal data on Indigenous health.

In Australia, established Indigenous cohort studies (including: Aboriginal Birth Cohort Study [7], Longitudinal Study of Indigenous Children [8], Study of Environment on Aboriginal Resilience and Child Health [9], Talking about the Smokes [10], Antecedents of Renal Disease in Aboriginal Children, Next Generation Youth Wellbeing Study [11]) have used field recruitment to sample from known populations in particular urban, regional, and remote areas. These field-based sampling approaches use purposive or strata-sampling with local Aboriginal or Torres Strait Islander data collectors who are employed or supported by partner Indigenous services. The face-to-face approach facilitates multiple options for completion of a survey, including self-completion; completion through interview; and, use of interpreters.

Aboriginal and Torres Strait Islander people comprise 3% of the Australian population, with a substantial proportion (19%) living in remote areas compared with 2% of non-Indigenous Australians living remotely [12]. These factors increase the complexities and resources required to recruit a national, heterogeneous sample that reflects the cultural, linguistic and geographic diversity of the population. All the cited Indigenous cohort studies are small-scale (*n* < 3000) and most have focussed on linguistically or culturally specific communities or geographic regions, presenting a substantial gap in large-scale, national longitudinal approaches to data collection among Aboriginal and Torres Strait Islander Australians.

Australian cohort studies have demonstrated that Aboriginal and Torres Strait Islander people do respond to postal surveys, though not always to the same extent as the total Australian population. The 45 and Up Study, involving adults aged ≥45 years from New South Wales (NSW), recruited 1939 Aboriginal participants through postal surveys (0.7% of overall cohort population compared to 2.8% of the NSW population). The 45 and Up survey sampled from the Medicare Australia database, which provides close to complete coverage of the total Australian population [13]. The 1999 Australian Women’s Health Survey (AWHS) found the proportion of Aboriginal and Torres Strait Islander women recruited from the postal survey was similar to the total Indigenous population (2.5%, *n* = 900) [14]. However, Aboriginal women living remotely were under-represented in the AWHS cohort, despite an over-sampling of remote areas [15]. These respective studies achieved an overall response rates between 18 and 44% in total sample population, but response rates specific to Indigenous participants were not calculated [15, 16]. In both studies, the Indigenous status of participants could only be identified post recruitment.

Until recently, it has been virtually impossible to determine a sampling frame to specifically distribute postal surveys to Aboriginal and Torres Strait Islander people. Telephone books and electoral rolls do not include Indigenous status information. Hospital records and other health registers do record Indigenous status, however data can: be unreliable due to poor recording of Indigenous status; be restricted due to privacy; be available only at state or territory-levels [17];, and include only the unwell population or birth population [18–20]. In 2006, Medicare added an Indigenous status question on enrolment and renewal forms [21]. This change has enabled researchers to request sampling of Indigenous persons from the Medicare Australia Enrolment Database. At October 2017, 533,832 people in the Medicare Australia Enrolment Database had indicated they were Aboriginal and/or Torres Strait Islander, covering ~ 60–70% of the total Indigenous population.

To our knowledge, Mayi Kuwayu, the National Study of Aboriginal and Torres Strait Islander Wellbeing is the first to test postal recruitment for an Indigenous cohort. The Study is a cohort of Aboriginal and Torres Strait Islander adults (≥16 years), designed to enable quantification of the relationships between health, culture and wellbeing [6]. The baseline sample is being recruited through a two-stage postal distribution: a preliminary distribution of 20,000 surveys and a main distribution of 180,000 surveys (total *N* = 200,000). The sample is supplemented with field recruitment. The aim of this cross-sectional analysis was to determine response rates to the preliminary postal distribution, overall and by age group, gender, level of remoteness and by jurisdiction, to inform the Study’s ongoing recruitment approaches.

## Methods

### Mayi Kuwayu study preliminary sample frame

Eligibility for receiving a Mayi Kuwayu survey in the post was defined by the following criteria: recorded as Aboriginal and/or Torres Strait Islander in the Medicare Enrolment Australia Database; and, aged 16 years or older. We used total Indigenous identified adults (≥16 years) in the Medicare Australia Enrolment Database to determine a stratified sample (*n* = 20,000) according to age, gender and remoteness (Table 1).

**Table 1:** Sampling frame for the Mayi Kuwayu Study preliminary postal distribution (*n* = 20,000), 30 October 2018

		Major cities	Regional	Remote	***Total***
**Females**					
16–24	Total Indigenous persons enrolled in Medicare^a^	15,600	21,300	9300	46,200
	% enrolled	4.6%	6.3%	2.8%	14.0%
	**Number sent the postal survey**	**928**	**1267**	**553**	**2748**
25–34	Total Indigenous persons enrolled in Medicare^a^	16,200	19,300	9800	45,300
	% enrolled	4.8%	5.7%	2.9%	13.5%
	**Number sent the postal survey**	**964**	**1148**	**583**	**2695**
35–49	Total Indigenous persons enrolled in Medicare^a^	13,500	16,900	10,300	40,700
	% enrolled	4.0%	5.0%	3.1%	12.1%
	**Number sent the postal survey**	**803**	**1005**	**613**	**2421**
≥ 50	Total Indigenous persons enrolled in Medicare^a^	14,900	19,300	10,000	44,200
	% enrolled	4.4%	5.7%	3.0%	13.1%
	**Number sent the postal survey**	**886**	**1148**	**595**	**2629**
**Males**					
16–24	Total Indigenous persons enrolled in Medicare^a^	13,800	19,800	8800	42,400
	% enrolled	4.1%	5.9%	2.6%	12.6%
	**Number sent the postal survey**	**821**	**1178**	**523**	**2522**
25–34	Total Indigenous persons enrolled in Medicare^a^	14,400	18,500	8600	41,500
	% enrolled	4.3%	5.5%	2.6%	12.3%
	**Number sent the postal survey**	**857**	**1101**	**512**	**2470**
35–49	Total Indigenous persons enrolled in Medicare^a^	11,900	15,900	9100	36,900
	% enrolled	3.5%	4.7%	2.7%	11.0%
	**Number sent the postal survey**	**708**	**946**	**541**	**2195**
≥ 50	Total Indigenous persons enrolled in Medicare^a^	12,700	17,100	9200	39,000
	% enrolled	4.0%	5.0%	3.0%	12.0%
	**Number sent the postal survey**	**756**	**1017**	**547**	**2320**
**Total**	Total Indigenous persons enrolled in Medicare^a^	113,000	148,100	75,100	336,200
	% enrolled	33.6%	44.1%	22.3%	100.0%
	**Number sent the postal survey**	**6723**	**8810**	**4467**	**20,000**

^**a**^Total Indigenous persons (rounded to the hundred) in Medicare Australia Enrolment Database, data provided by Department of Health on 19 June 2018

The Department of Human Services (DHS) mailed surveys to individuals, randomly selected from the total pool of eligible persons in each age-sex-remoteness stratum. The survey pack included: a prepaid return envelope; an eight-page coloured survey; and, an information sheet. DHS required use of a standard government cover letter and outer envelope. The letter informed recipients that DHS was sending the information on behalf of the research team. No addresses or names were provided to the research team. In addition to the paper survey, respondents were provided options to complete the survey online or via a free-call helpline. The survey packs were mailed on 30 October 2018.

### Survey respondent eligibility

An eligible survey respondent for inclusion in this analysis was defined by: (i) aged 16 years or older; and, (ii) responded to the Mayi Kuwayu postal survey in the preliminary phase (30 October 2018–11 February 2019). Paper surveys with IDs between 100,000 and 200,000 were included, as this was within the range allocated for postal distribution. Online survey responses were included if respondents selected “sent to me in the post” or “sent to someone I know” when answering the survey question, “How did you find out about the survey?” All surveys completed in field-based recruitment were excluded.

### Variables

We calculated age from reported date of birth and date of survey completion, and recoded to age groups 16–24, 25–34, 35–49 and ≥ 50 years. If the date of survey completion was missing, the age was calculated at 11 February 2019 (the cut-off point for the preliminary phase). Gender was a self-reported survey item and classified as female, male or other. Reported postcode of residence was used to define the Remoteness Areas based on Australian Geographical Standard Classification (AGSC), which categorises areas as ‘major cities’, ‘inner regional’, ‘outer regional’, ‘remote’ or ‘very remote’ [22]. Remoteness Areas were grouped: major cities, regional (inner and outer regional), remote (remote and very remote). For postcodes mapped on to multiple Remoteness Areas, we assigned the Remoteness Area with the highest proportion of population, as determined by ABS [23].

We used other survey variables (education, financial status and first language) to characterise respondents. Education level was derived from the highest level of completed education and included the categories: no schooling, up to Year 10, Year 12, technical/trade and university qualification. Household financial status was defined from the question “What words describe your family’s money situation”? Responses include: 1“We have a lot of savings”, 2“We have some savings”, 3“We have just enough to get us to the next payday”, 4“We run out of money before payday”, 5“We are spending more than we get”, 6“Unsure”. These were grouped as “Savings” [1, 2], “Just enough” [3], “No savings” [4, 5], Unsure [6]. First language was defined as: “Aboriginal or Torres Strait Islander language”, “English” or “other”. We generated a new variable to represent mode of completion (online versus paper survey) based on data from the survey provider.

### Statistical analysis

The response rate was defined as the number of returned postal surveys divided by the total number of surveys sent, according to the sample frame (Table 1), overall and within each stratum (age, gender, remoteness). We analysed response rates to the postal distribution at two time points (4 weeks and 15 weeks). We separately calculated response rates based on jurisdiction (state or territory). We report 95% confidence intervals for response rates assuming a binomial distribution. We summarised the number of responses over time (by week) and by response mode (paper survey, online).

The respondents were characterised according to age, gender, remoteness, education level, financial status and first language, and we report numbers and percentages. All missing or unclassifiable data were reported and listed as ‘unclassifiable’. We compared response mode (online versus paper) across sociodemographic characteristics of respondents using Fisher’s exact test. We used the strata-level response-rate results at 4 weeks to inform the main-stage postal sample for the Mayi Kuwayu Study (*N* = 180,000). We compared results at 4 weeks to 15 weeks to assess if response rates were sensitive to time. Analysis was undertaken in STATA version 15.

To gain insight into the extent of surveys that did not reach the intended respondent, we reviewed data on return to senders provided by the DHS. In addition, we reviewed reasons for non-completion of the postal survey from Mayi Kuwayu Study helpline callers and, field researcher reports on reasons why people who received the postal survey did not respond.

### Approval and ethics

Ethics approvals were granted for the conduct of the Study from national and state/territory Human Research Ethics Committees. Internal governmental approvals (Department of Health and DHS) were obtained to post surveys specifically to Aboriginal and Torres Strait Islander people enrolled in Medicare.

## Results

### Response rates to Mayi Kuwayu preliminary phase postal distribution

A total of 465 individuals responded to the preliminary Mayi Kuwayu postal distribution. One participant was under the age of 16 years, seven participants were non-Indigenous and one participant completed the survey twice, the duplicate response and non-eligible participants were excluded from the analysis (465–9 = 456). The overall response rate was 2.3% (n/*N* = 456/20000). Response rates were highest in major cities (2.9, 95%CI:2.5–3.3) and substantially lower in remote areas (0.8%, 0.6–1.1). There were similar response rates between men and women (male 2.1%, 1.8–2.4, versus female 2.4%, 2.1–2.7). Older age groups were more likely to respond than younger age groups (Table 2).

**Table 2:** Response rate to the Mayi Kuwayu Study preliminary postal distribution (*N* = 20,000), overall and by gender, age group and remoteness

	Major cities			Regional			Remote			Total		
	n/N	response rate (%)	95%CI	n/N	response rate (%)	95%CI	n/N	response rate (%)	95%CI	n/N	response rate (%)	95%CI
**Male**												
16–24	8/821	1.0	0.4–1.9	5/1178	0.4	0.1–1.0	2/523	0.4	< 0.1–1.4	16/2522	0.6	0.4–1.0
25–34	13/857	1.5	0.8–2.6	9/1101	0.8	0.4–1.5	3/512	0.6	0.1–1.7	25/2470	1.0	0.7–1.5
35–49	15/708	2.1	1.2–3.5	13/946	1.4	0.7–2.3	1/541	0.2	< 0.1–1.0	31/2195	1.4	1.0–2.0
≥ 50	45/756	6.0	4.4–7.9	61/1017	6.0	4.6–7.6	11/547	2.0	1.0–3.6	117/2320	5.0	4.2–6.0
*All male*	*84/3142*	*2.7*	*2.1–3.3*	*90/4242*	*2.1*	*1.7–2.6*	*18/2123*	*0.8*	*0.5–1.3*	*195/9507*	*2.1*	*1.8–2.4*
**Female**												
16–24	23/928	2.5	1.6–3.7	24/1267	1.9	1.2–2.8	2/553	0.4	< 0.1–1.3	49/2748	1.8	1.3–2.4
25–34	16/964	1.7	1.0–2.7	15/1148	1.3	0.7–2.1	1/583	0.2	< 0.1–1.0	32/2695	1.2	0.8–1.7
35–49	21/803	2.6	1.6–4.0	16/1005	1.6	0.9–2.6	2/613	0.3	< 0.1–1.2	39/2421	1.6	1.1–2.2
≥ 50	49/886	5.5	4.1–7.2	71/1148	6.2	4.9–7.7	12/595	2.0	1.0–3.5	132/2629	5.0	4.2–5.9
*All female*	*109/3581*	*3.0*	*2.5–3.7*	*128/4568*	*2.8*	*2.3–3.3*	*18/2344*	*0.8*	*0.5–1.2*	*255/10493*	*2.4*	*2.1–2.7*
**TOTAL**	**194/6723**	**2.9**	**2.5–3.3**	**222/8810**	**2.5**	**2.2–2.9**	**37/4467**	**0.8**	**0.6–1.1**	**456/20000**	**2.3**	**2.1–2.5**

*18 participants were missing one or more variable of interest (gender, remoteness, age group) or reported “other” to gender. These missing/other data are not presented in the stratified results but are included in the totals

The highest response rates were obtained in strata levels aged ≥50 years: males from regional areas 6.0% (4.6–7.6), males from major cities 6.0% (4.4–7.9), females from major cities 5.5% (4.1–7.2), and females from regional areas 6.2% (4.9–7.7) (Table 2). Response rates were ≥ 2.5% in these jurisdictions: Australian Capital Territory (6.3%,3.1–11.3), New South Wales (3.3%,2.8–3.8), Tasmania (5.0%,3.4–7.1), Victoria (3.6%, 2.7–4.7) and < 2% in Queensland (1.7%,1.4–2.9), Western Australia (1.2%,0.8–1.6), Northern Territory (0.6, 0.3–1.1) (Table 3).

**Table 3:** Response rate to the Mayi Kuwayu Study preliminary postal distribution (*N* = 20,000), by State/Territory

State/Territory	Total Responses (n)	Total surveys mailed (N)	Response rate	95%CI
**Australian Capital Territory**	10	159	6.3%	3.1–11.3
**New South Wales**	179	5415	3.3%	2.8–3.8
**Northern Territory**	12	1904	0.6%	0.3–1.1
**Queensland**	110	6318	1.7%	1.4–2.1
**South Australia**	25	1044	2.4%	1.6–3.5
**Tasmania**	30	598	5.0%	3.4–7.1
**Victoria**	53	1468	3.6%	2.7–4.7
**Western Australia**	37	3094	1.2%	0.8–1.6
**Total**	**456**	**20,000**	**2.3%**	**2.1–2.5**

### Responses over time and by response mode

**Fig. 1:**
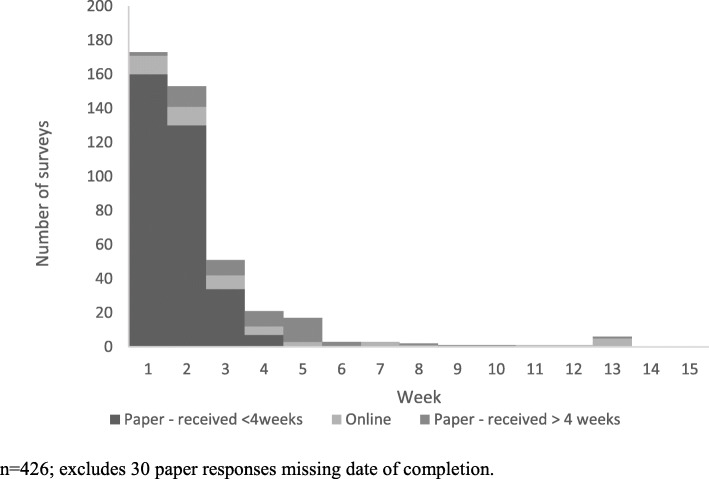
Responses to the Mayi Kuwayu preliminary postal distribution by week and response mode. *n* = 426; excludes 30 paper responses missing date of completion

Most surveys were received by the end of the fourth week (*n* = 390, 85.5%). Sixty-six (14.5%) postal surveys were received between four and 15 weeks (Table 4); however, a large percentage of the paper survey responses (51.6%, *n* = 32/53) received in this period were completed by the respondent within the initial four-week period (Fig. 1).

**Table 4:** Characteristics of preliminary Mayi Kuwayu Study postal respondents, overall and by response mode

	Respondents	Response mode		
	N	% Paper (n/N)	% Online (n/N)	*p*-value
**TOTAL**	456	89.5 (408/456)	10.5 (48/456)	–
**Response received**				
Within 4 weeks	390	91.0 (355/390)	9.0 (35/390)	0.02
> 4 to 14 weeks	66	80.3 (53/66)	19.7 (13/66)	
**Gender**				
Male	195	89.7 (175/196)	10.3 (20/196)	1.0
Female	255	89.4 (228/255)	10.6 (27/255)	
*Unclassifiable*	*6*	*83.3(5/6)*	*16.7(1/6)*	
**Remoteness**				
Major cities	194	85.6 (166/194)	14.4 (28/194)	0.06
Regional	222	92.8 (206/222)	7.2 (16/222)	
Remote	37	89.2 (33/37)	10.8 (4/37)	
*Unclassifiable*	*3*	*100(3/3)*	*0(0/3)*	
**Age group**				
16–24	65	84.6 (55/65)	15.4 (10/65)	< 0.01
25–34	58	81.0 (47/58)	19.0 (11/58)	
35–49	70	82.9 (58/70)	17.1 (12/70)	
≥ 50	251	94.8 (238/252)	5.2 (13/252)	
*Unclassifiable*	*12*	*83.3(10/12)*	*16.7(2/12)*	
**Education**				
No school	2	100 (2/2)	0 (0/2)	0.04
Up to Year 10	189	94.2 (178/189)	5.8 (11/189)	
Year 12	51	94.1 (48/51)	5.9 (3/51)	
Technical or trade	126	87.3 (110/126)	12.7 (16/126)	
University	77	83.1 (64/77)	16.9 (13/77)	
*Unclassifiable*	*11*	*54.5(6/11)*	*45.5(5/11)*	
**Financial status**				
Some savings	212	89.6 (190/212)	10.4 (22/212)	0.51
Just enough	144	91.0 (131/144)	9 (13/144)	
No savings	60	88.3 (53/60)	11.7 (7/60)	
Unsure	20	100 (20/20)	0 (0/20)	
*Unclassified*	*20*	*70.0(14/20)*	*30.0(6/20)*	
**First language**				
Indigenous language	9	66.7 (6/9)	33.3 (3/9)	< 0.01
English	429	90.9 (390/430)	9.1 (39/430)	
Other	9	33.3 (3/9)	66.7 (6/9)	
*Unclassifiable*	*9*	*100.0(9/9)*	*0(0/9)*	

*the category unclassifiable was not included in fisher exact test

Most participants responded on the paper survey (*n* = 408, 89.5%). Online responses were more common among: younger respondents (15.4% among 16–24 years, 19.0% among 25–34 years, and 17.1% among 35–49 years) compared to respondents ≥50 years (5.2%, *p*-value for fisher’s exact test< 0.01); those with higher education levels (16.9% of university educated) compared to those with lower levels (5.8% of those with year 10 or lower education, p-value 0.04); and, respondents whose first language was an Indigenous language (33.3%) or other (66.6%) compared to those whose first language was English (9.3%, *p*-value< 0.01). Online responses were also more common in participants who responded after 4 weeks (20.7%) compared with those who responded in the first 4 weeks (9.0%).

### Respondent characteristics to the preliminary postal distribution

Respondents included 255 females (55.9%) and 195 males (42.8%), with 194 respondents from major cities (42.5%), 222 respondents from regional areas (48.7%), and 36 respondents from remote areas (8.1%) (Table 4). Among age groups, 251 respondents were aged ≥50 (55.0%), 70 respondents were 35–49 (15.4%), 58 respondents were 25–34 (12.7%), and 65 respondents were 16–24 (14.3%). The survey respondents represented a diversity of financial and education levels. Few respondents spoke an Aboriginal and Torres Strait Islander language as their first language (2.0%, *n* = 9).

### Reasons for non-completion, ineligibility and return to senders

DHS estimated receiving 500 ‘returns to sender’ (exact number not known), with these surveys destroyed according to a departmental protocol. Sixty-eight calls to the Study helpline were recorded over the period, including from 40 non-Indigenous people who received the survey and were ineligible to participate. Several Indigenous callers who received the survey in the post were concerned about a perceived link between government and the Study, including concerns about data ownership (*n* = 12). Other callers indicated that they could not complete the survey due to disability and/or low literacy level (*n* = 5). All invitations for the caller to complete the survey over the phone were declined. One person who received the postal survey was also contacted in the field recruitment over this period and had not completed the survey due to low literacy.

### Determining the sampling frame for the second stage postal distribution

The response rates at 4 weeks (Supplementary Table 1) were used to determine the distribution for the next stage of postal distribution (180,000 surveys). There was no material difference in response rates at 4 weeks and 15 weeks in each stratum of the sample (Supplementary Table 2). The sampling approach which had been initially proposed for the Study, prior to consideration of these results, planned to over-sample lower responding groups to achieve 500 responses per strata and maximise sample heterogeneity. However, the results from this preliminary phase demonstrated this was not feasible. For example, males 16–24 years living in major cities had a response rate of 0.6%. This equates to receiving one response per 167 surveys posted; to receive 500 returned surveys, an estimated 83,500 surveys would need to be sent, exceeding the number of Indigenous males enrolled in the Medicare database in this stratum (*n* = 12,800 males, major cities, 16–24 years).

The Study team decided that it was a more pragmatic and efficient use of resources to mail the remaining 180,000 surveys to eligible participants in the high response rate groups (Table 5). Compared to sampling all groups proportionately, over-sampling the high response rate groups increased the expected postal survey returns from ~ 4000 (2.3% response rate) to ~ 5500 (3.1% response rate). Updated Aboriginal and Torres Strait Islander Medicare enrolment figures were provided by DHS on 8 February 2019 to determine the strategy for distributing the remaining 180,000 postal surveys (Table 5).

**Table 5:** Sample for Mayi Kuwayu Study second phase postal distribution and expected number of surveys returned based on preliminary response rate

	Major cities			Regional			Remote			**TOTAL**		
	Response rate (%)	Surveys sent (N)	Estimated returns (n)	Response rate (%)	Surveys sent (N)	Estimated returns (n)	Response rate (%)	Surveys sent (N)	Estimated returns (n)	Response rate (%)	Surveys sent (N)	Estimated returns (n)
**Male**												
16–24	Not surveyed											
25–34	Not surveyed											
35–49	2.1	10,660	224	1.4	12,176	170				1.7	22,836	388
≥ 50	6.0	10,351	621	6.0	13,409	805	2.0	5044	101	5.0	28,804	1440
**Female**												
16–24	2.5	13,950	349	1.9	18,020	342				2.1	31,970	671
25–34	1.7	15,640	266	1.3	17,605	229				1.5	33,245	499
35–49	2.6	12,660	329	1.6	15,293	245				2.0	27,953	559
≥ 50	5.5	12,943	712	6.2	16,031	994	2.0	6122	122	5.0	35,096	1775
**TOTAL**	**3.3**	**76,204**	**2515**	**3.1**	**92,534**	**2869**	**2.0**	**11,166**	**223**	**3.1**	**179,904**	**5577**

## Discussion

Based on these response rates and a revised postal sampling strategy, we estimated a return of 5900 postal surveys to the Mayi Kuwayu Study, forming the largest Aboriginal and Torres Strait Islander cohort to date. The preliminary postal distribution returned 456 eligible surveys achieving a 2.3% overall response rate, which is lower than rates in other national surveys for the total Australia population and our estimated response rate of 8–20% [6]. There was approximately a 6.0% response rate in the four of the strata levels group (males ≥50 regional, Males ≥50 major cities, Women ≥50 regional, Women ≥50 major cities). The trends are consistent with other Australian cohort studies that have found older age groups and less remote participants are more likely to respond [15]; however, our results contrast with these earlier studies as we did not observe a significant difference in response rate by gender. There was higher uptake of the online survey completion option by younger age groups compared with older age groups, which is also consistent with a recent qualitative study of Indigenous Australians that suggested older participants prefer paper surveys to online [24].

Explanations of non-response are likely to encompass commonly known barriers in Indigenous health research. Indigenous people have described these, including: a history of exploitative research; feeling over-researched; having competing priorities; concerns about government data collection and control; financial barriers; and, not using a participant’s first language and associated literacy and numeracy issues [25–27]. There is no national data on English literacy for the adult Aboriginal and Torres Strait Islander population; however, literacy rates overall are lower than the total Australian population, particularly for people in remote areas [28]. One survey of Aboriginal adults in the Northern Territory (the state where we observed the lowest response rate, 0.6%) reported that 63% (*n* = 554/660) of participants believed their English literacy skills were inadequate for everyday life [29]. There may be a preference for face-to-face rather than postal survey by some Indigenous participants as this can support: participation of people with low numeracy and literacy; the use of interpreters; and, the use Indigenous researchers to engage participants [6].

The response rate could have been affected by a large quantity of surveys not reaching recipients. We could only rely on the DHS estimate of ‘returns to sender’ (*n* ~ 500). Earlier research has demonstrated counts of ‘returns to sender’ underestimate the number of surveys not received [14]. Further, in this context high levels of mobility among Indigenous people in some regions may intensify this issue leading to inaccurate addresses in the database [30, 31]. In order to improve estimates, we have commissioned DHS to provide exact ‘return to sender’ counts at the strata level in the follow-up postal distribution. This will help quantify the proportion of all surveys that did not reach an eligible participant and identify the demographic groups where ‘return to sender’ is most prevalent and may be particularly hard to reach.

### Implications of non-response for recruitment approaches

The lower response rates than expected required the Study team to revise the postal sampling strategy to maximise the number of completed surveys and increase efficiency. If the intended minimum baseline of 16,000 respondents is still to be achieved, it will likely require intensification of field recruitment. Depending on response to the next phase, field recruitment numbers may surpass postal recruitment as the primary recruitment method for the baseline cohort.

Prior evidence has demonstrated that low response rates do not necessarily cause non-respondent bias [32–34] and highlighted the importance of collecting as much demographic and population characteristic data as possible. Collection of this type of data enables testing of a specific substantiative analysis and consideration of sample heterogeneity. Mealing et al. found that internal validity in cohort studies is supported by ensuring that there is adequate variation in exposures, a more important factor to consider than response rates [6]. The choice to target specific age groups and regions through the postal survey may have consequences on the heterogeneity of the sample and sub-group analysis, and importantly could reduce the cultural and/or linguistic diversity of the sample.

Field-based recruitment in the Mayi Kuwayu Study will target groups that are under-represented in the postal survey (i.e. younger age groups, remote areas, language groups, prison populations). Focused field-based recruitment can also enhance regional or finer analysis, supporting much needed analysis of community level results and variation in exposures and outcomes between regions [35]. Analysis of the final baseline cohort compared to the total Aboriginal and Torres Strait Islander population is planned.

### Improving recruitment approaches

A number of studies have investigated methods for improving response rates to surveys [3, 32, 36, 37]. One systematic review suggested the response rates can be improved by using: reminder and follow-up letters, incentives, television advertising, field recruitment and shorter survey options [37]. Attempts to increase recruitment since the preliminary phase include: television advertising; increased field recruitment; and, increased social media advertising. Other improvements were not possible for the Mayi Kuwayu Study, including: using reminder and follow-up letters (survey materials were sent by DHS and the research team did not have access to addresses); incentivising participation (due to resource and ethical constraints); and, shortening the survey (because this was fixed at the start of the Study). Extensive piloting of the survey substantially reduced the survey length; however, estimates indicate it takes 30 min to 1 h.

If increased attention is given to field recruitment for the baseline, then some review of these methods could be beneficial in achieving a more heterogeneous sample. The Study has supported different sampling approaches for field recruitment based on community preferences, including convenience; snowballing; strata; and, community-based population; sampling approaches. The dispersed population in regional and remote areas mean that field-based sampling can occur in clusters of communities and regions. These approaches do not necessarily allow for the calculation of response rates and requires more complicated processes to apply population weights and/or adjust for geographic clustering [8]. Where community requests for population level data are made, then application of strata-sampling and cluster sampling approaches (such as the approach used in ABS sampling) may support weighted data for those communities [38]. However, weighting of the total baseline sample may not be possible.

### Analysis limitations and strengths

This is the first analysis of responses rates to a national postal survey of Aboriginal and Torres Strait Islander adults and provides new insights on recruitment approaches for large-scale Indigenous cohort studies. An important strength of this analysis is the value to informing ongoing recruitment approaches in our Study and with Indigenous peoples generally. The initial stage has allowed the opportunity to consider issues of heterogeneity in the sample and non-response bias. Further research in the Mayi Kuwayu Study and other Indigenous cohort studies will continue to assess the cultural appropriateness of different recruitment approaches. Our finding that respondents were more likely to use an online platform when their first language was an Indigenous language compared with respondents who spoke English as a first language is one potential area. It will be important to determine whether these respondents are also living in remote areas given the lower response rates in remote areas where Indigenous language is most likely to be spoken as a first language.

A limitation of our study was the use of postcodes to determine level of remoteness, as ~ 17.3% (79/456) of postcodes in this sample crossed boundaries, and may mean that some respondents’ remoteness area was incorrectly coded. Geocoding of address data, rather than the use of postcodes, in future analysis would reduce misclassification. Further, this analysis included those who responded, “sent to someone I know” (*n* = 26/456 respondents). We considered this a postal response, although these respondents may differ in terms of age, gender and remoteness from the intended recipient (the sample frame). We could not account for these differences in our analysis. This limitation does not influence the overall response rate. It may have minor implications on response rates at the strata level (6% of all responses were “sent to someone I know”). In any cases, our response rates could be underestimated, given the known surveys that went to ineligible participants, were returned to sender or did not reach their intended recipient.

## Conclusion

To improve the heterogeneity of Indigenous cohorts, we have determined that a combination of postal and field recruitment approaches remain important methods for recruitment of Aboriginal and Torres Strait Islander adults. In our study, the postal responses rates may have been reduced because of the substantial (19%) proportion of the total Aboriginal and Torres Strait Islander population living in remote areas (who are more likely to converse in their Indigenous language), concerns relating to the government cover letter, socioeconomic status, levels of English literacy and negative experiences of research. The Study’s next recruitment phase was adapted to sample from the highest responding strata by post, increase field recruitment in remote areas and among younger age groups and expand media promotion.

## Supplementary information

**Additional file 1: Table S1.** Mayi Kuwayu preliminary phase postal response rates at 4 weeks, 28 November 2018. **Table S2.** Difference in Mayi Kuwayu preliminary phase response rates at 4 weeks and 15 weeks, strata with highest respond rates are shaded

## Data Availability

The Mayi Kuwayu data is governed by a group of Aboriginal and Torres Strait Islander leaders. Request can be submitted to this group for data access.

## Abbreviations

ABS: Australian Bureau Statistics
DHS: Department of Human Service
CI: Confidence interval
