# Supplementary material for: Who responds? An examination of response rates to a national postal survey of Aboriginal and Torres Strait Islander adults, 2018-2019
Source: BMC Med Res Methodol. 2020 Jun 10;20:149. doi: 10.1186/s12874-020-00970-8 (PMC7285609; doi:10.1186/s12874-020-00970-8)
Supplement: Supplementary file 1 — Additional file 1: Table S1. Mayi Kuwayu preliminary phase postal response rates at 4 weeks, 28 November 2018. Table S2. Difference in Mayi Kuwayu preliminary phase response rates at 4 weeks and 15 weeks, strata with highest respond rates are shaded [file 12874_2020_970_MOESM1_ESM.docx]

**Supplementary Table 1: Mayi Kuwayu preliminary phase postal response rates at 4 weeks, 28 November 2018**

|  | **Response at 4 weeks, 30 October 2018 – 28 November 2018** | | | | | | | | | | | |
| --- | --- | --- | --- | --- | --- | --- | --- | --- | --- | --- | --- | --- |
|  | **Major cities** | | | **Regional** | | | **Remote** | | | **TOTAL** | | |
|  | **n/N** | **Response rate (%)** | **95% CI** | **n/N** | **Response rate (%)** | **95% CI** | **n/N** | **Response rate (%)** | **95% CI** | **n/N** | **Response rate (%)** | **95% CI** |
| **Male** |  |  |  |  |  |  |  |  |  |  |  |  |
| 16-24 | 6/821 | 0.7 | 0.3–1.6 | 5/1178 | 0.4 | 0.1–1.0 | 2/523 | 0.4 | <0.1–1.4 | 14/2522 | 0.6 | 0.3–0.9 |
| 25-34 | 8/857 | 0.9 | 0.4–1.8 | 7/1101 | 0.6 | 0.3–1.3 | 2/512 | 0.4 | <0.1–1.4 | 17/2470 | 0.7 | 0.4–1.1 |
| 35-49 | 14/708 | 2.0 | 1.1–3.3 | 11/946 | 1.2 | 0.6–2.1 | 1/541 | 0.2 | <0.1–1.0 | 27/2195 | 1.2 | 0.8–1.8 |
| ≥50 | 39/756 | 5.2 | 3.7–7.0 | 53/1017 | 5.2 | 3.9–6.8 | 11/547 | 2.0 | 1.0–3.6 | 103/2320 | 4.4 | 3.6–5.4 |
| All male | 70/3142 | 2.2 | 1.7–2.8 | 77/4242 | 1.8 | 1.4–2.3 | 17/2123 | 0.8 | 0.5–1.3 | 166/9507 | 1.7 | 1.5–2.0 |
| **Female** |  |  |  |  |  |  |  |  |  |  |  |  |
| 16-24 | 17/928 | 1.8 | 1.1–2.9 | 23/1267 | 1.8 | 1.2–2.7 | 2/553 | 0.4 | <0.1–1.3 | 42/2748 | 1.5 | 1.1–2.1 |
| 25-34 | 12/964 | 1.2 | 0.6–2.2 | 14/1148 | 1.2 | 0.7–2.0 | 1/583 | 0.2 | <0.1–1.0 | 27/2695 | 1.0 | 0.7–1.5 |
| 35-49 | 19/803 | 2.4 | 1.4–3.7 | 14/1005 | 1.4 | 0.8–2.3 | 1/613 | 0.2 | <0.1–0.9 | 34/2421 | 1.4 | 1.0–2.0 |
| ≥50 | 44/886 | 5.0 | 3.6–6.6 | 59/1148 | 5.1 | 3.9–6.6 | 10/595 | 1.7 | 0.8–3.1 | 113/2629 | 4.3 | 3.6–5.1 |
| All female | 92/3581 | 2.6 | 2.1–3.1 | 111/4568 | 2.4 | 2.0-2.9 | 15/2344 | 0.6 | 0.4–1.1 | 218/10493 | 2.1 | 1.8–2.4 |
| **TOTAL** | **167/6723** | **2.5** | **2.1–2.9** | **194/8810** | **2.2** | **1.9–2.5** | **33/4467** | **0.7** | **0.5-1.0** | **390/20000** | **2.0** | **1.8–2.2** |

*18 participants were missing one or more variable of interest (gender, remoteness, age group) or reported “other” to gender. These missing/other data are not presented in the stratified results but are included in the totals.

**Supplementary Table 2: Difference in Mayi Kuwayu preliminary phase response rates at 4 weeks and 15 weeks**, strata with highest respond rates are shaded

|  | **Major cities** | | | | | **Regional** | | | | | **Remote** | | | | |
| --- | --- | --- | --- | --- | --- | --- | --- | --- | --- | --- | --- | --- | --- | --- | --- |
|  |  |  |  |  |  |  |  |  |  |  |  |  |  |  |  |
|  | **Response rate at 4 weeks** | **95%CI** | **Response rate at 15 weeks** | **95%CI** | **15wk–4wk** | **Response rate at 4 weeks** | **95%CI** | **Response rate at 15 weeks** | **95%CI** | **15wk–4wk** | **Response rate at 4 weeks** | **95%CI** | **Response rate at 15 weeks** | **95%CI** | **15wk–4wk** |
| **Male** |  |  |  |  |  |  |  |  |  |  |  |  |  |  |  |
| 16-24 | 0.7 | 0.3–1.6 | 1.0 | 0.4–1.9 | 0.3 | 0.4 | 0.1–1.0 | 0.4 | 0.1–1.0 | 0 | 0.4 | <0.1–1.4 | 0.4 | <0.1–1.4 | 0 |
| 25-34 | 0.9 | 0.4–1.8 | 1.5 | 0.8–2.6 | 0.6 | 0.6 | 0.3–1.3 | 0.8 | 0.4–1.5 | 0.2 | 0.4 | <0.1–1.4 | 0.4 | 0.1–1.7 | 0 |
| 35-49 | 2.0 | 1.1–3.3 | 2.1 | 1.2–3.5 | 0.1 | 1.2 | 0.6–2.1 | 1.4 | 0.7–2.3 | 0.2 | 0.2 | <0.1–1.0 | 0.2 | <0.1–1.0 | 0 |
| ≥50 | 5.2 | 3.7–7.0 | 6.0 | 4.4–7.9 | 0.8 | 5.2 | 3.9–6.8 | 6.0 | 4.6–7.6 | 0.8 | 2.0 | 1.0–3.6 | 2.0 | 1.0–3.6 | 0 |
| All male | 2.1 | 1.7–2.8 | 2.7 | 2.1–3.3 | 0.6 | 1.8 | 1.4–2.3 | 2.1 | 1.7–2.6 | 0.3 | 0.8 | 0.5–1.3 | 0.8 | 0.5–1.3 | 0 |
| **Female** |  |  |  |  |  |  |  |  |  |  |  |  |  |  |  |
| 16-24 | 1.8 | 1.1–2.9 | 2.5 | 1.6–3.7 | 0.7 | 1.8 | 1.2–2.7 | 1.9 | 1.2–2.8 | 0.1 | 0.4 | <0.1–1.3 | 0.4 | <0.1–1.3 | 0 |
| 25-34 | 1.2 | 0.6–2.2 | 1.7 | 1.0–2.7 | 0.5 | 1.2 | 0.7–2.0 | 1.3 | 0.7–2.1 | 0.1 | 0.2 | <0.1–1.0 | 0.2 | <0.1–1.0 | 0 |
| 35-49 | 2.4 | 1.4–3.7 | 2.6 | 1.6–4.0 | 0.2 | 1.4 | 0.8–2.3 | 1.6 | 0.9–2.6 | 0.2 | 0.2 | <0.1–0.9 | 0.3 | <0.1–1.2 | 0.1 |
| ≥50 | 5.0 | 3.6–6.6 | 5.5 | 4.1–7.2 | 0.5 | 5.1 | 3.9–6.6 | 6.2 | 4.9–7.7 | 1.1 | 1.7 | 0.8–3.1 | 2.0 | 1.0–3.5 | 0.3 |
| All female | 2.6 | 2.1–3.1 | 3.0 | 2.5–3.7 | 0.4 | 2.4 | 2.0-2.9 | 2.8 | 2.3–3.3 | 0.2 | 0.6 | 0.4–1.1 | 0.8 | 0.5–1.2 | 0.2 |
| **TOTAL** | 2.5 | 2.2–3.1 | 2.9 | 2.5–3.3 | 0.4 | 2.2 | 1.9–2.5 | 2.5 | 2.2–2.9 | 0.3 | 0.7 | 0.5-1.0 | 0.8 | 0.6–1.1 | 0.1 |
